# Osteocytes, not Osteoblasts or Lining Cells, are the Main Source of the RANKL Required for Osteoclast Formation in Remodeling Bone

**DOI:** 10.1371/journal.pone.0138189

**Published:** 2015-09-22

**Authors:** Jinhu Xiong, Marilina Piemontese, Melda Onal, Josh Campbell, Joseph J. Goellner, Vladimir Dusevich, Lynda Bonewald, Stavros C. Manolagas, Charles A. O’Brien

**Affiliations:** 1 Center for Osteoporosis and Metabolic Bone Diseases, University of Arkansas for Medical Sciences, Little Rock, Arkansas, United States of America; 2 The Central Arkansas Veterans Healthcare System, Little Rock, Arkansas, United States of America; 3 Department of Oral Biology, University of Missouri-Kansas City School of Dentistry, Kansas City, Missouri, United States of America; Faculté de médecine de Nantes, FRANCE

## Abstract

The cytokine receptor activator of nuclear factor kappa B ligand (RANKL), encoded by the *Tnfsf11* gene, is essential for osteoclastogenesis and previous studies have shown that deletion of the *Tnfsf11* gene using a *Dmp1-Cre* transgene reduces osteoclast formation in cancellous bone by more than 70%. However, the *Dmp1-Cre* transgene used in those studies leads to recombination in osteocytes, osteoblasts, and lining cells making it unclear whether one or more of these cell types produce the RANKL required for osteoclast formation in cancellous bone. Because osteoblasts, osteocytes, and lining cells have distinct locations and functions, distinguishing which of these cell types are sources of RANKL is essential for understanding the orchestration of bone remodeling. To distinguish between these possibilities, we have now created transgenic mice expressing the Cre recombinase under the control of regulatory elements of the *Sost* gene, which is expressed in osteocytes but not osteoblasts or lining cells in murine bone. Activity of the *Sost-Cre* transgene in osteocytes, but not osteoblast or lining cells, was confirmed by crossing *Sost-Cre* transgenic mice with *tdTomato* and *R26R* Cre-reporter mice, which express tdTomato fluorescent protein or LacZ, respectively, only in cells expressing the Cre recombinase or their descendants. Deletion of the *Tnfsf11* gene in *Sost-Cre* mice led to a threefold decrease in osteoclast number in cancellous bone and increased cancellous bone mass, mimicking the skeletal phenotype of mice in which the *Tnfsf11* gene was deleted using the *Dmp1-Cre* transgene. These results demonstrate that osteocytes, not osteoblasts or lining cells, are the main source of the RANKL required for osteoclast formation in remodeling cancellous bone.

## Introduction

RANKL is essential for osteoclast formation, function, and survival [1]. RANKL is also important for many other processes, such as lymphocyte differentiation, mammary gland development, microfold cell production in the gut, and thermoregulation in females [2–5]. Consistent with these diverse functions, RANKL is expressed by a variety of different cell types and in response to many different stimuli [6]. We and others have shown previously that crossing mice with a conditional *Tnfsf11* allele (hereafter referred to as *Tnfsf11-f*) with mice harboring a *Dmp1-Cre* transgene increases bone mass as early as 2 months of age and that this is associated with very low osteoclast number in cancellous bone [7;8]. Based on these results and on the observation that the *Dmp1-Cre* transgene leads to efficient deletion of loxP-flanked sequences in osteocytes, we concluded that osteocytes are an essential source of the RANKL required for osteoclast formation in cancellous bone.

In the course of our work, we noted that the *Dmp1-Cre* transgene also leads to efficient recombination in matrix synthesizing osteoblasts, which was detected using Cre-reporter mice [7]. Subsequently, a similar finding was independently reported [9]. Most osteoblasts die by apoptosis at the end of the bone formation process and the remaining cells become one of two distinct cell types [10]. Some of them are buried within the bone matrix and become osteocytes. The remaining osteoblasts flatten out to become lining cells covering the quiescent bone surface. Since the *Dmp1-Cre* transgene we used causes recombination in osteoblasts, and since lining cells are derived from osteoblasts, it is likely that the *Tnfsf11* gene was deleted from both osteoblasts and lining cells, as well as osteocytes, in *Dmp1-Cre;Tnfsf11-f/f* mice.

In our previous study we had reasoned that a contribution of RANKL by osteoblasts is unlikely based on two pieces of evidence [7]. First, depletion of osteoblasts by pre-treating mice with osteoprotegerin (OPG) for two weeks, which suppresses both osteoclast and osteoblast number due to coupling [11], did not alter either basal or parathyroid hormone (PTH)-stimulated RANKL levels in bone. Second, deletion of the *Tnfsf11* conditional allele using an *Osx1-Cre* transgene that was not activated until after 4 months of age, and therefore caused deletion in only a small subset of osteocytes but should have led to efficient deletion in osteoblasts, did not alter osteoclast number in cancellous bone. However, it was not possible to confirm deletion of RANKL from osteoblasts in vivo in the *Osx1-Cre;Tnfsf11-f/f* mice. Thus, the first piece of evidence was indirect and the second assumed efficient recombination in osteoblasts. In addition, work of others published subsequently contends that osteoblasts, but not osteocytes, are the major source of RANKL for cancellous osteoclast formation [12–14].

The goal of the present study was to develop mice in which Cre recombination occurs in osteocytes but not in osteoblasts or lining cells and then use these mice to more specifically address the contribution of osteocyte RANKL to osteoclast formation. The *Sost* gene, encoding the Wnt antagonist sclerostin, is expressed in osteocytes but not in osteoblasts or lining cells [15]. Therefore, we generated transgenic mice that express Cre recombinase under the control of *Sost* gene regulatory elements and demonstrated that the transgene leads to recombination in osteocytes but not osteoblasts or lining cells. Moreover, deletion of the *Tnfsf11* gene using *Sost-Cre* mice produced a skeletal phenotype indistinguishable from that caused by deletion using the *Dmp1-Cre* transgene, supporting our conclusion that osteocyte-derived RANKL is essential for osteoclast formation in cancellous bone.

## Materials and Methods

### Animal studies

Tg(Sost[RP24-276023]-GFP/Cre) transgenic mice, hereafter referred to as *Sost-Cre* mice, were generated by insertion, via recombineering [16], of an internal ribosome entry site (IRES)—green fluorescent protein (GFP)::Cre recombinase fusion protein into the second exon of the murine *Sost* gene located within a bacterial artificial chromosome (BAC), clone RP24-276023. The IRES-GFP::Cre cassette was inserted in such way that it replaced most of the *Sost* 3’ untranslated region. Full-length BAC DNA was microinjected into fertilized eggs from C57BL/6 mice and four independent founder lines were obtained. Each line displayed similar patterns of Cre recombination as measured histologically with the Cre-reporter strain R26R [17]. One line was selected for use in all studies described herein. Generation of mice harboring the *Tnfsf11-f* allele has been described previously [7] and the *Tnfsf11-f* mice used in this study were on a mixed C57BL/6 and 129/sv genetic background. Conditional knockout mice for this study were obtained using a 2-step breeding strategy. First, hemizygous *Sost-Cre* transgenic mice were crossed with heterozygous *Tnfsf11-f* mice to generate heterozygous *Tnfsf11-f* offspring with and without a *Sost-Cre* allele. These offspring were then intercrossed to generate the following types of offspring: wild-type mice, mice hemizygous for the *Sost-Cre* allele, mice homozygous for the *Tnfsf11*-f allele, and mice homozygous for the *Tnfsf11*-f allele that were also hemizygous for the *Sost-Cre* allele. Offspring were genotyped by PCR using the following primer sequences: Cre-for, 5’-GCGGTCTGGCAGTAAAAACTATC-3’, Cre-rev, 5’-GTGAAACAGCATTGCTGTCACTT-3’, product size 102 bp; RANKL-flox-for, 5’-CTGGGAGCGCAGGTTAAATA-3’, RANKL-flox-rev, 5’-GCCAATAATTAAAATACTGCAGGAAA-3’, product size 108 bp (wild-type) and 251 bp (floxed allele). Generation of the 8 kb and 10 kb *Dmp1-Cre* transgenic mice [18;19], *R26R* mice [17], and *tdTomato* reporter mice (line Ai9) [20], has been described previously. In all animal studies, mice were euthanized via CO_2_ inhalation from a tank source at a displacement rate of from 10% to 30% volume/minute until all movement ceased followed by an additional 1 minute in the chamber. Euthanasia was confirmed by lack of reflex to corneal touching. All animal studies were approved by the Institutional Animal Care and Use Committee of the University of Arkansas for Medical Sciences.

### Bone mineral density (BMD) determination

BMD was determined by dual energy x-ray absorptiometry (DXA) using a PIXImus densitometer (GE-Lunar Corp, Madison, WI) and software version 2.0. BMD was measured at three sites. The total body window was defined as the whole body image minus the calvarium, mandible, and teeth. Except for the first few caudal vertebrae, the tail was not included. The spine window was a rectangle that included all lumbar vertebrae. The femoral window captured the right femur. Scan acquisition time was four minutes. The mice were sedated with isoflurane inhalation during scanning to keep them motionless for the required four minutes and to facilitate rapid post examination recovery. The animals were monitored by observation of the righting reflex, respiration, and heart rate. Using a proprietary skeletal phantom, the total body BMD was measured over the past 5 years with a mean coefficient of variation of 0.40%.

### μCT analysis

The femurs and lumbar vertebrae 4 (L4) were dissected, cleaned of soft tissues, fixed in Millonig’s formalin, and gradually dehydrated into 100% ethanol. Bones were loaded into 12.3 mm diameter scanning tubes and imaged in a μCT (model μCT40, Scanco Medical, Wayne, PA). The scans were integrated into 3-D voxel images (1024 x 1024 pixel matrices for each individual planar stack) and a Gaussian filter (sigma = 0.8, support = 1) was used to reduce signal noise. A threshold of 200 was applied to all scans at medium resolution (E = 55 kVp, I = 145 μA, integration time = 200ms). Whole vertebrae were scanned with a transverse orientation excluding any bone outside the vertebral body. The cortical bone and the primary spongiosa were manually excluded from the analysis. All trabecular measurements were made by drawing contours every 10 to 20 slices and voxel counting was used for bone volume per tissue volume and sphere filling distance transformation indices, without pre-assumptions about the bone shape as a rod or plate for trabecular microarchitecture. Cortical thickness was measured at the femoral mid-diaphysis. Calibration and quality control were performed weekly using five density standards and spatial resolution was verified monthly using a tungsten wire rod. We based beam-hardening correction on the calibration records. We made corrections for 200 mg hydroxyapatite for all energies. The mean coefficient of variation of the μCT phantom (performed weekly) during the conduct of these studies was 1.23%.

### RNA purification and gene expression analysis

Calvariae and the L5 vertebrae were dissected from animals, cleaned of muscle, frozen immediately in liquid nitrogen, and stored at -80°C. Soft tissues were dissected from animals, frozen immediately in liquid nitrogen, and stored at -80°C. The distal and proximal ends of the tibiae were removed and bone marrow cells were flushed out completely with PBS. Total RNA was purified from tissues using Ultraspec reagent (Biotecx Laboratories, Houston, TX), according to the manufacturer’s instructions. Three μg of total RNA was reverse-transcribed into cDNA as using the High Capacity Reverse Transcriptase kit (Life Technologies, Grand Island, NY). For quantitating the *Sost-Cre* transgene expression, RNA was purified using DNA free RNA kit (Zymo research, Irvine, CA) and a minus reverse transcriptase control was used in the subsequent quantitative RT-PCR setup. Taqman quantitative RT-PCR was performed using the following Taqman assays from Life Technologies: *Tnfsf11* (Mm00441908_m1); *Sost-Cre* transgene (custom Taqman assay number AJRR802); mitochondrial ribosomal protein S2 (*Mrps2*) (for, 5’-CCCAGGATGGCGACGAT-3’, rev, 5’-CCGAATGCTGTAATGGCGTAT-3’, probe, 5’-FAM-TCCAGAGCAGGATCC-NFQ-3’); and *Actb* (Mm00607939_s1). Relative mRNA levels were calculated by normalizing to the house-keeping gene *Mrps2* or *Actb* using the ΔCt method [21].

### Genomic DNA isolation from osteocyte-enriched bone

The distal and proximal ends of the femur were removed and bone marrow cells were flushed out completely with PBS. The surfaces of the bone shafts were scraped with a scalpel to remove the periosteum and then cut into a few small pieces. Bone pieces were then digested with 1 ml of Hank’s solution containing 1 mM CaCl_2_ and 1 mg/ml of collagenase (type I:II, ratio 1:3) (Worthington Biochemical Corporation, Freehold, NJ) in a 12-well-plate. A total of 6 digestions for 15 minutes each were performed at 37°C in a water bath shaker to remove the cells on the bone surface. After the final digestion, bone pieces were decalcified in 14% EDTA for 1 week. Decalcified bone was then digested with proteinase K (0.5 mg/ml in 10 mM Tris, pH 8.0, 100 mM NaCl, 20 mM EDTA, and 1% SDS) at 55°C overnight. Genomic DNA was then isolated by phenol/chloroform extraction and ethanol precipitation. Two custom Taqman assays were obtained from Life Technologies for quantifying *Tnfsf11* gene deletion efficiency: one specific for sequences between the loxP sites (for, 5’-GCCAGTGGACTTACTCAAACCTT-3’; rev, 5’-GGTAGGGTTCAACTGAAGGGTTTA-3’; probe, 5’-FAM-CCTCCTCCTCATGGTTTAGT-NFQ-3’) and the other specific for sequences downstream from the 3’ loxP site (for, 5’-GGTGCCGTGCATTATCCTAGAC-3’; rev, 5’-AAGTAATGTGACCCTTGGAGAACTG-3’; probe, 5’-FAM-CTAGCACACGTGCCTGCT-NFQ-3’).

### Histology of decalcified bone

Femurs and L1-L3 vertebrae were fixed for 24 h in 10% Millonig’s formalin, decalcified in 14% EDTA for 1 week, embedded in paraffin, and then 5 μm longitudinal sections were cut. After removal of paraffin and rehydration, the sections were stained for tartrate-resistant acid phosphatase (TRAP) activity and counterstained with toluidine blue. To visualize cartilage, sections were stained with safranin-O and counter-stained with fast green. Quantitative histomorphometry to determine osteoclast number was performed on the TRAP-stained sections using a computer and digitizer tablet (OsteoMetrics, Decatur, GA) interfaced to a Zeiss Axioscope (Carl Zeiss, Thornwood, NY) equipped with a drawing tube. In L1-L3 vertebrae, all cancellous bone was analyzed with the exception of the region within two visual fields of the growth plates and any trabeculae that were in direct contact with cortical bone. In femurs, cancellous bone analysis was performed beginning two visual fields below the growth plate and extended the entire remaining area of cancellous bone in the samples from *Tnfsf11-f/f* mice. Since the cancellous bone of the conditional knockout mice was much more extensive, a region similar in area to the *Tnfsf11-f/f* controls was measured and averaged approximately 3 mm^2^. Histological measurements of the endocortical bone of the femur also began two visual fields below the growth plate and were continued to the diaphysis, with the diaphysis defined as the midway point between the ends of the femur.

### Histology of non-decalcified bone

Mice were injected with calcein (20 mg/kg body weight) 7 and 3 days before euthanization. Femurs for 5-bromo-4-chloro-3-indolyl-β-D-galactopyranoside (X-gal) staining were fixed in fresh 0.2% glutaraldehyde at 4°C for 7 days and then were immersed in 30% sucrose for a minimum of 1 day and then embedded in Cryo-Gel (Electron Microscopy Sciences, Hatfield, PA) for frozen sectioning. Five micron sections were obtained using a Leica cryostat with tape transfer system (Leica Microsystems LM3050S, Buffalo Grove, IL). X-gal staining was performed on frozen sections at room temperature for 6 hours in staining solution (1 mg/ml X-gal, 5 mM potassium ferrocyanide, 5 mM potassium ferricyanide, 2 mM MgCl_2_, 0.01% sodium deoxycholate, 0.02% NP40, and 20 mM Tris-HCl, pH 7.4). After washing with wash buffer (2 mM Mgcl_2_, 0.01% sodium deoxycholate, 0.02% NP40, and 20 mM Tris-HCl, pH 7.4) for 15 minutes, sections were mounted with aqueous mounting medium for imaging. For visualization of tdTomato protein, femurs were dissected, cleaned of soft tissue, and then fixed in 4% paraformaldehyde overnight at 4°C. After fixation, femurs were soaked in 30% sucrose for a minimum of 1 day and then processed from cryo-sectioning as above. Five micron sections were mounted with Vectashield mounting medium containing 4',6-diamidino-2-phenylindole (DAPI) (Vector laboratory Inc., Burlingame, CA). Sections were then observed using an Olympus BX53 microscope with a DAPI/FITC/TXRED triple filter (Olympus, Center Valley, PA) to simultaneously detect calcein labels, tdTomato fluorescence, and nuclei. High magnification images were obtained using a Zeiss LSM510 Meta confocal microscope (Zeiss, Oberkochen, Germany).

### Transmission electron microscopy (TEM)

For X-gal staining, vertebrae from 2-month-old mice were dissected into small pieces and fixed with a solution of 2% paraformaldehyde and 2.5% glutaraldehyde at 4°C for 6 hours. After fixation, samples were decalcified in 14% Tris-buffered EDTA for 2 weeks at 4°C and then were stained in X-gal staining solution at room temperature in the dark for 36 hours. Samples were washed three times in H_2_O for 5 minutes and post-fixed in 2% paraformaldehyde and 2.5% glutaraldehyde in 0.1 M cacodylate buffer overnight at 4°C. Samples were treated with 1% osmium tetroxide for 1.5 hours, dehydrated in graded solutions of ethanol (33%, 67%, 85%, 95%, 100%) followed with 1:1 solution of ethanol and propylene oxide, pure propylene oxide and finally with 1:1 solution of propylene oxide and epoxy resin (Embed-812, Electron Microscopy Sciences, Hatfield, PA). After final infiltration with pure epoxy resin, specimens were incubated in at 60° Celsius for 48 hours. Ultrathin sections were cut with an EM UC7 ultramicrotome (Leica Microsystems Inc., Buffalo Grove, IL); to preserve better contrast from X-gal depositions no additional stains (i.e. uranyl acetate and lead citrate) were applied to sections. Specimens were observed with a CM12 electron microscope (FEI, Hillsboro, OR) at 80 kV accelerating voltage. Micrographs were acquired with an Orius 832 CCD camera (Gatan, Pleasanton, CA).

### Flow cytometry

Spleen cells and bone marrow cells from the femur were isolated and stained in PBS containing 3% FBS. Samples were blocked with anti-CD16/CD32 for 5 minutes and then stained for 30 minutes using hematopoietic cell panel antibodies (CD11b, CD3, CD19, CD220, and Ter119). All antibodies were purchased from BD Biosciences (San Jose, CA). After washing to remove unbound antibodies, samples were analyzed by flow cytometry using a BD FACS Aria flow cytometer (BD Biosciences). The data were analyzed using Flowjo Software (FlowJo, LLC, Ashland, OR). Appropriate gates for the cell populations were drawn with guidance of Fluorescence Minus One (FMO) controls (BD Biosciences).

### Statistics

Student’s *t*-test or one-way ANOVA were used to detect statistically significant effects, after determining that the data were normally distributed and exhibited equivalent variances. In some cases, log transformation was used to obtain normally-distributed data. All *t*-tests were two-sided. Holm-Sidak corrections were used for multiple comparisons. P-values less than 0.05 were considered as significant. Values in all figures represent means ± s.d.

## Results

### Generation of *Sost-Cre* transgenic mice

We have demonstrated previously that a *Dmp1-Cre* transgene harboring approximately 10 kb of 5’-flanking region, hereafter referred to as *10 kb Dmp1-Cre*, is active in osteocytes as well as in matrix synthesizing osteoblasts [7]. Since lining cells are derived from osteoblasts, it is likely that the *10 kb Dmp1-Cre* transgene also causes recombination in lining cells. To identify a Cre-driver strain that is active in osteocytes but not osteoblasts or lining cells, we examined the activity of a different *Dmp1-Cre* transgene, which harbors a shorter region of 5’-flanking region and will hereafter be referred to as *8 kb Dmp1-Cre* [18]. To do this, *8 kb Dmp1-Cre* transgenic mice were crossed with *R26R* Cre-reporter mice [17]. *10 kb Dmp1-Cre* mice were used as positive control. X-gal staining of non-decalcified femur sections from 2-month-old mice showed that the *8 kb* and *10 kb Dmp1-Cre* transgenes were both active in osteocytes and in cells on the endocortical and cancellous bone surfaces (S1 Fig). Incorporation of calcein label showed co-localization of these blue cells with mineralizing bone surface (S1 Fig), demonstrating that the X-gal positive cells were matrix synthesizing osteoblasts. Therefore, both the *8 kb* and *10 kb Dmp1-Cre* transgenic models lead to recombination in osteoblasts and lining cells.

**Fig 1:**
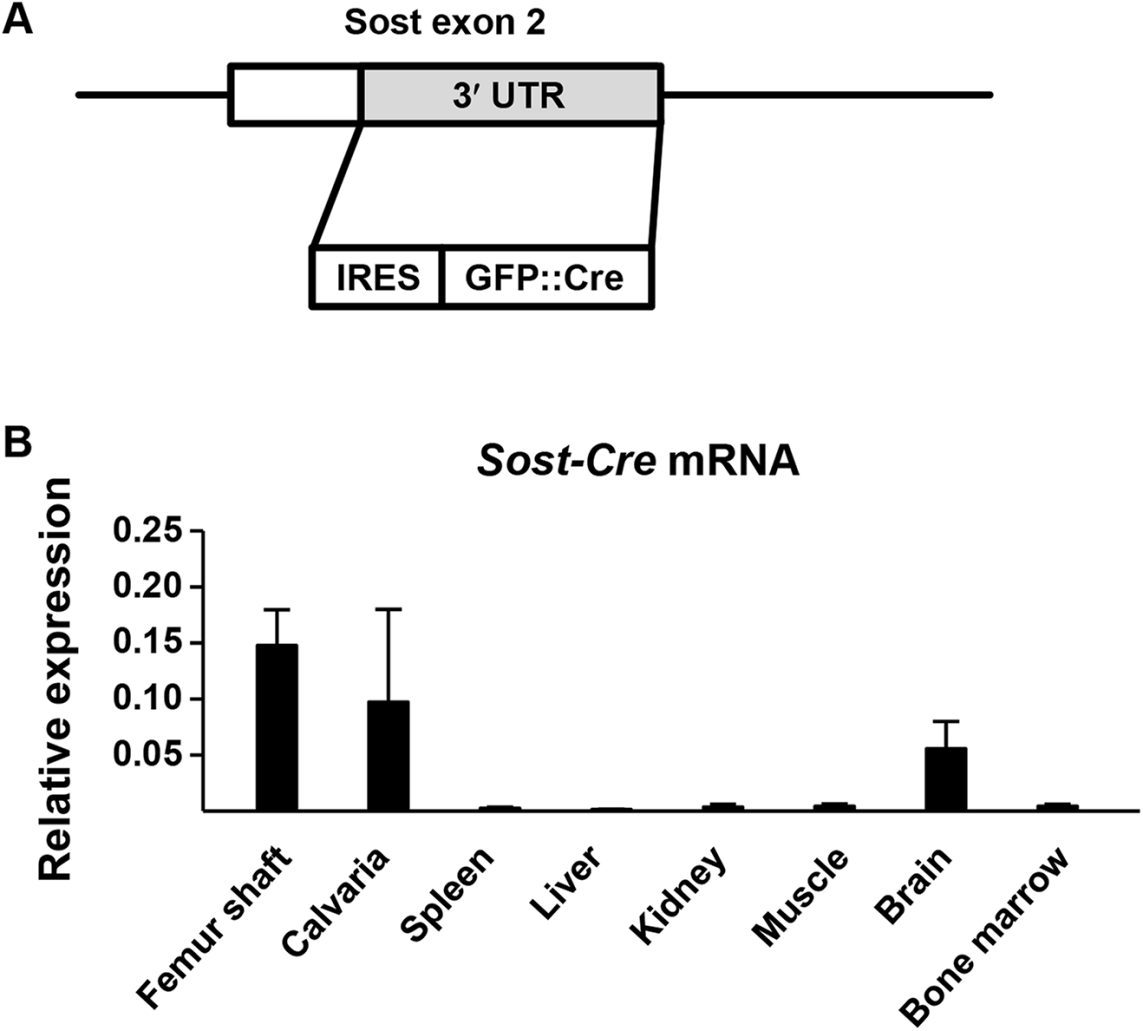
Generation of *Sost-Cre* transgenic mice. (A) Diagram of a portion of the *Sost-Cre* transgene construct used to generate *Sost-Cre* transgenic mice. UTR, untranslated region, IRES, internal ribosome entry site, GFP::Cre, fusion protein of GFP and the Cre recombinase. (B) *Sost-Cre* transgene mRNA levels in different tissues of 6-week-old mice male mice as measured by quantitative RT-PCR. n = 4 animals for each tissue.

Having established that the two different *Dmp1-Cre* transgenes cause recombination in both osteocytes and osteoblasts, we next sought to generate a novel Cre-deleter strain that would lead to recombination in osteocytes but not osteoblasts or lining cells. To do this, we generated transgenic mice that express Cre recombinase under the control of regulatory elements of the murine *Sost* gene by inserting Cre coding sequence into exon 2 of the *Sost* gene contained in a bacterial artificial chromosome (Fig 1A). The expression level of the *Sost-Cre* transgene in different tissues was determined by quantitative real time PCR and revealed that it is highly expressed in femoral bone shafts, calvaria, and brain, but not in spleen, liver, kidney, muscle, or bone marrow cells (Fig 1B).

### The *Sost-Cre* transgene causes recombination in osteocytes but not in osteoblasts

**Fig 2:**
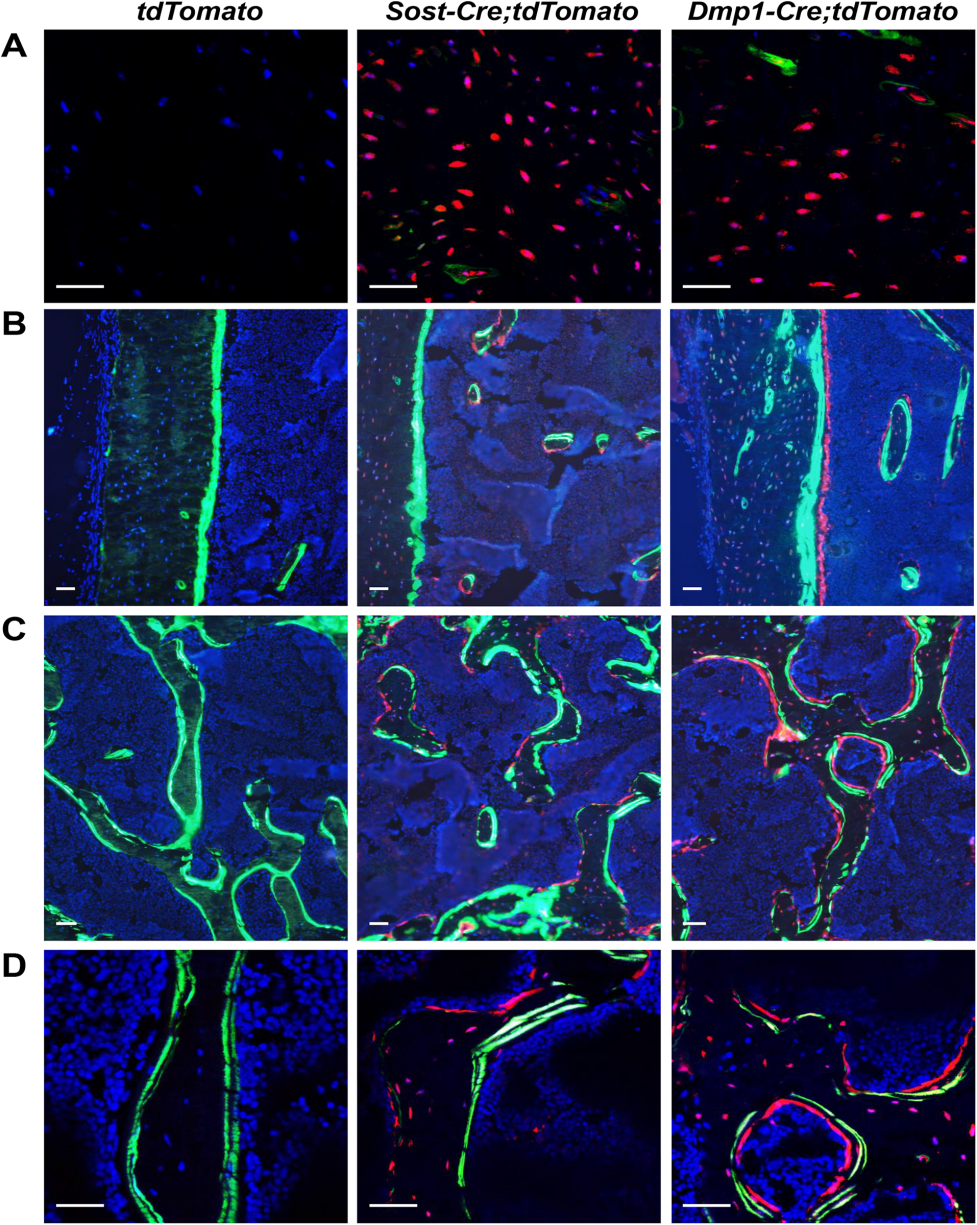
Anaylsis of *Sost-Cre* transgenic mice using *tdTomato* Cre-reporter mice. Fluorescent images of frozen histological sections of femurs from 2-month-old *tdTomato* control, *Sost-Cre;tdTomato*, and *10 kb Dmp1-Cre;tdTomato* mice injected with calcein. (A) Confocal image of osteocytes in cortical bone. (B) Epifluorescent image of endocortical surface. (C) Epifluorescent image of cancellous bone. (D) Higher magnification of the cancellous bone from (C). Scale bar, 50 μm. Green: calcein labeling of mineralizing bone; Red: cells that express tdTomato fluorescent protein upon Cre-mediated recombination.

To determine the activity of the *Sost-Cre* transgene in bone cells, we crossed *Sost-Cre* mice with *tdTomato* Cre-reporter mice. For comparison, *10 kb Dmp1-Cre* mice were also crossed with *tdTomato* mice. *tdTomato* mice express tdTomato fluorescent protein under the control of a ubiquitously-expressed promoter only after Cre-mediated removal of a loxP-flanked stop cassette [20]. Frozen sections of femurs from 2-month-old *10 kb Dmp1-Cre;tdTomato* mice confirmed that the *10 kb Dmp1-Cre* transgene led to recombination in osteocytes as well as osteoblasts on the endocortical and cancellous bone surfaces (Fig 2A–2D). The identity of the red cells on the bone surface as osteoblasts was demonstrated by co-localization with green calcein labels, which are indicative of sites of recent or ongoing bone formation. In contrast, the *Sost-Cre* transgene led to recombination in osteocytes but not in osteoblasts, as demonstrated by the lack of red fluorescent cells that co-localized with green calcein labels (Fig 2A–2D). However, we did observe red fluorescent cells on bone surfaces that were not on top of bone labeled by calcein. TRAP staining and fluorescent imaging of the same sections of the femur of *Sost-Cre;tdTomato* mice revealed that the red fluorescent cells were also TRAP-positive, demonstrating that they are osteoclasts (S2 Fig). In contrast, fluorescent red cells on bone surface of *Dmp1-Cre;tdTomato* mice were TRAP-negative (S2 Fig).

**Fig 3:**
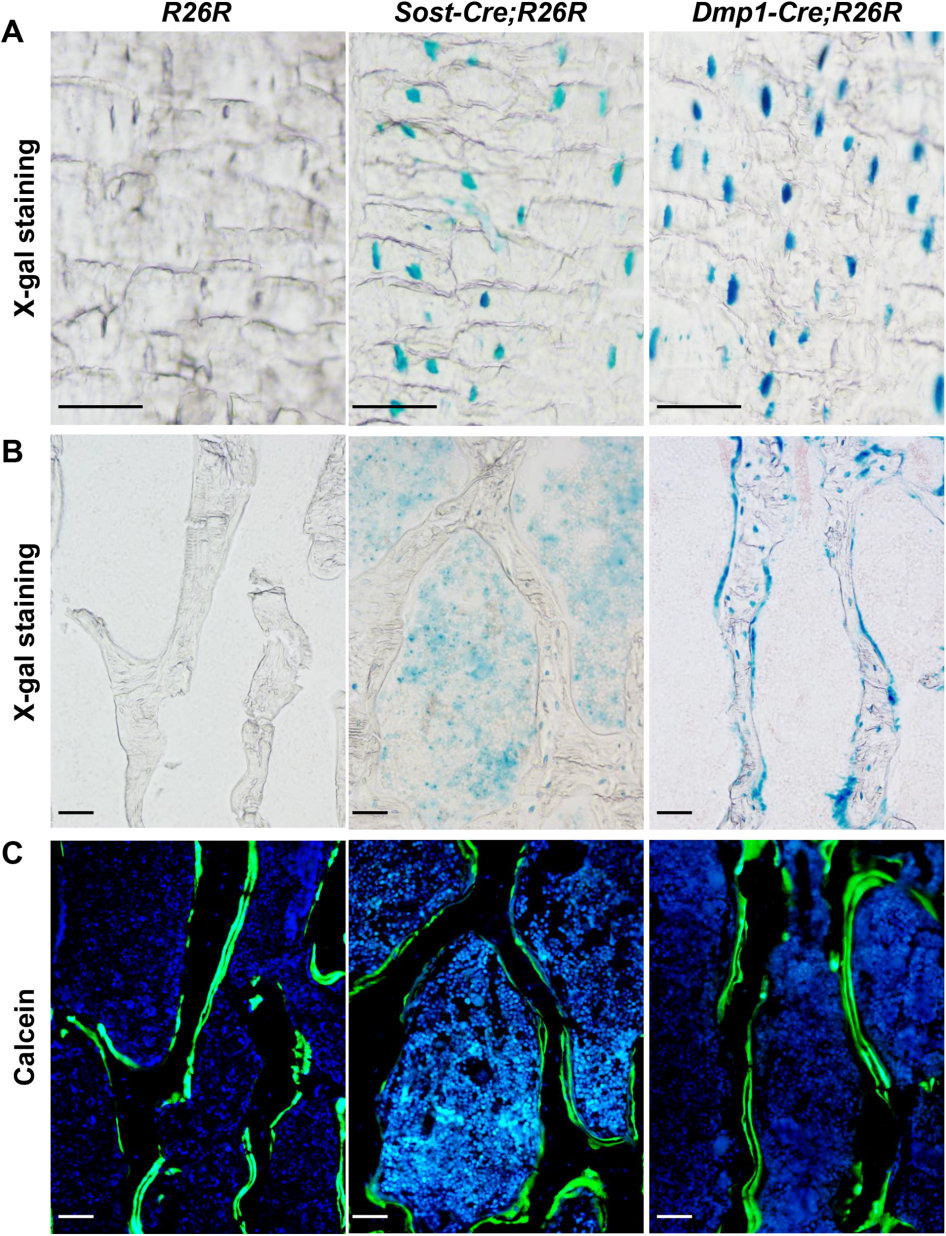
Analysis of *Sost-Cre* transgenic mice using *R26R* Cre-reporter mice. (A) Brightfield microscopy images of X-gal stained frozen histological sections of cortical bone and (B) cancellous bone in the femur of 2-month-old *R26R*, *Sost-Cre;R26R*, and *10 kb Dmp1-Cre;R26R* mice. (C) Epifluorescent image of the same cancellous bone as in (B) showing calcein labeling. Scale bar, 50 μm.

**Fig 4:**
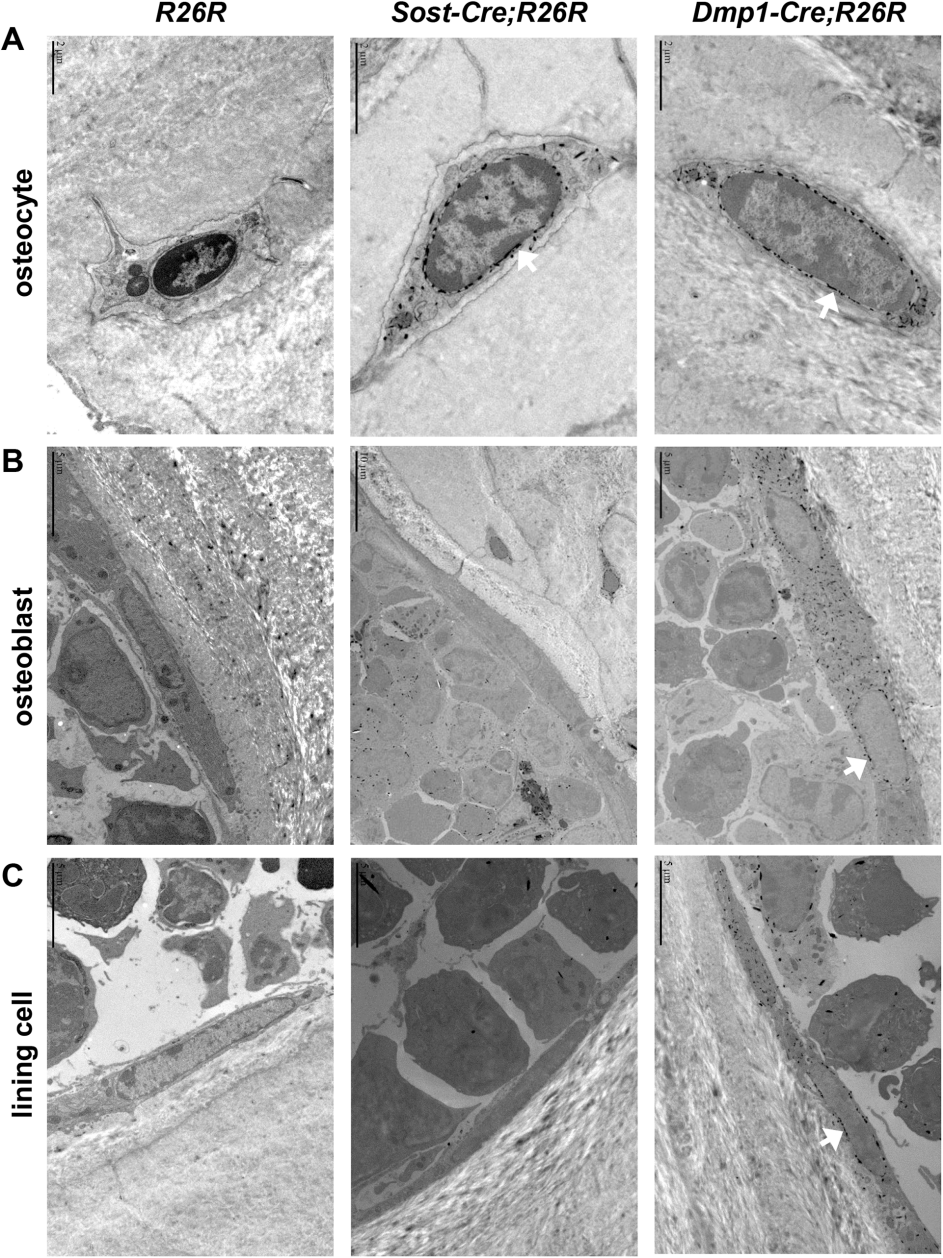
Analysis of *Sost-Cre* transgene activity by transmission electron microscopy. Transmission electron microscopy (TEM) images of electron-dense X-gal deposits in osteocytes (A), osteoblasts (B), and lining cells (C) on the cancellous bone surface of vertebra of 2-month-old *R26R*, *Sost-Cre;R26R*, and *10 kb Dmp1-Cre;R26R* mice. Size bars are present in each image, with the size indicated.

The Cre coding sequence used to create *Sost-Cre* mice is fused to the coding sequence of *GFP*. However, no GFP fluorescence was observed in any cell types in bone sections of these mice. Sequence analysis of the transgene revealed a single nucleotide change in the GFP sequence, resulting in an amino acid change from leucine to histidine at position 179 that may have disrupted GFP activity. Nonetheless, no mutations were detected in the Cre coding sequence and analyses with Cre-reporter strains revealed functional Cre activity by the transgene (Figs 2–4).

We noted that large numbers of red fluorescent cells were present in the bone marrow of *Sost-cre;tdTomato* mice (Fig 2B), suggesting that the *Sost-Cre* transgene might lead to recombination in cells of hematopoietic lineage. Flow cytometric analysis of bone marrow cells of *Sost-Cre;tdTomato* mice showed that 89% of bone marrow cells express red fluorescent protein (S3 Fig). Within the red fluorescent cell population, 12% were CD119+ erythrocytes, 3% were CD3+ T lymphocytes, 40% were CD 11b+ monocytes, and 24% were CD19+ B lymphocytes (S3 Fig). The ratio of these cell types to one another is similar to the ratio of these cell types in whole bone marrow (S3 Fig), suggesting that the *Sost-Cre* transgene is active in an early hematopoietic progenitor. Consistent with this idea, we were unable to detect expression of the endogenous *Sost gene* in CD19^+^ B cells, CD3^+^ T cells, CD11B^+^ monocytes, or mature osteoclasts (data not shown).

### The *Sost-Cre* transgene does not cause recombination in lining cells

Although the *Sost-Cre* transgene is active in hematopoietic cells, we reasoned that it would still be useful for distinguishing between the role of RANKL in osteocytes versus osteoblasts or lining cells if it does not lead to recombination in either of these latter two cell types. This is because we and others have shown that the source of the RANKL involved in cancellous bone remodeling is a cell type targeted by the *10 kb Dmp1-Cre* transgene, which does not lead to recombination in hematopoietic cells. Therefore, we next determined whether the *Sost-Cre* transgene is active in lining cells. Lining cells are very thin (less than 5 μm) and flattened against the bone surface and in mice are clearly distinguished from other cell types only at magnifications possible by electron microscopy. The insoluble blue precipitate formed by X-gal staining can be detected by transmission electron microscopy (TEM) as dark crystals that predominate around the nucleus [22;23]. Therefore, we crossed *Sost-Cre* and *10 kb Dmp1-Cre* mice with *R26R* reporter mice and prepared bone sections for both bright field microscopy and TEM. Bright field examination of X-gal-stained non-decalcified frozen sections of the femur confirmed that the *Sost-Cre* transgene is active in osteocytes but not in osteoblasts (Fig 3A–3C). Consistent with the observations made by light microscopy, TEM revealed X-gal precipitates localized around the nucleus of osteocytes but not osteoblasts or lining cells in sections from *Sost-Cre;R26R* mice (Fig 4A–4C). In contrast, X-gal precipitates were present in osteocytes, as well as osteoblasts and lining cells in *10 kb Dmp1-Cre;R26R* mice (Fig 4A–4C). Importantly, X-gal precipitate was not observed in *R26R* control mice (Fig 4A–4C). These results demonstrate that, in contrast to *Dmp1-Cre* transgenes, the *Sost-Cre* transgene leads to recombination in osteocytes but not osteoblasts or lining cells.

### Deletion of the *Tnfsf11* gene using the *Sost-Cre* transgene increases bone mass

**Fig 5:**
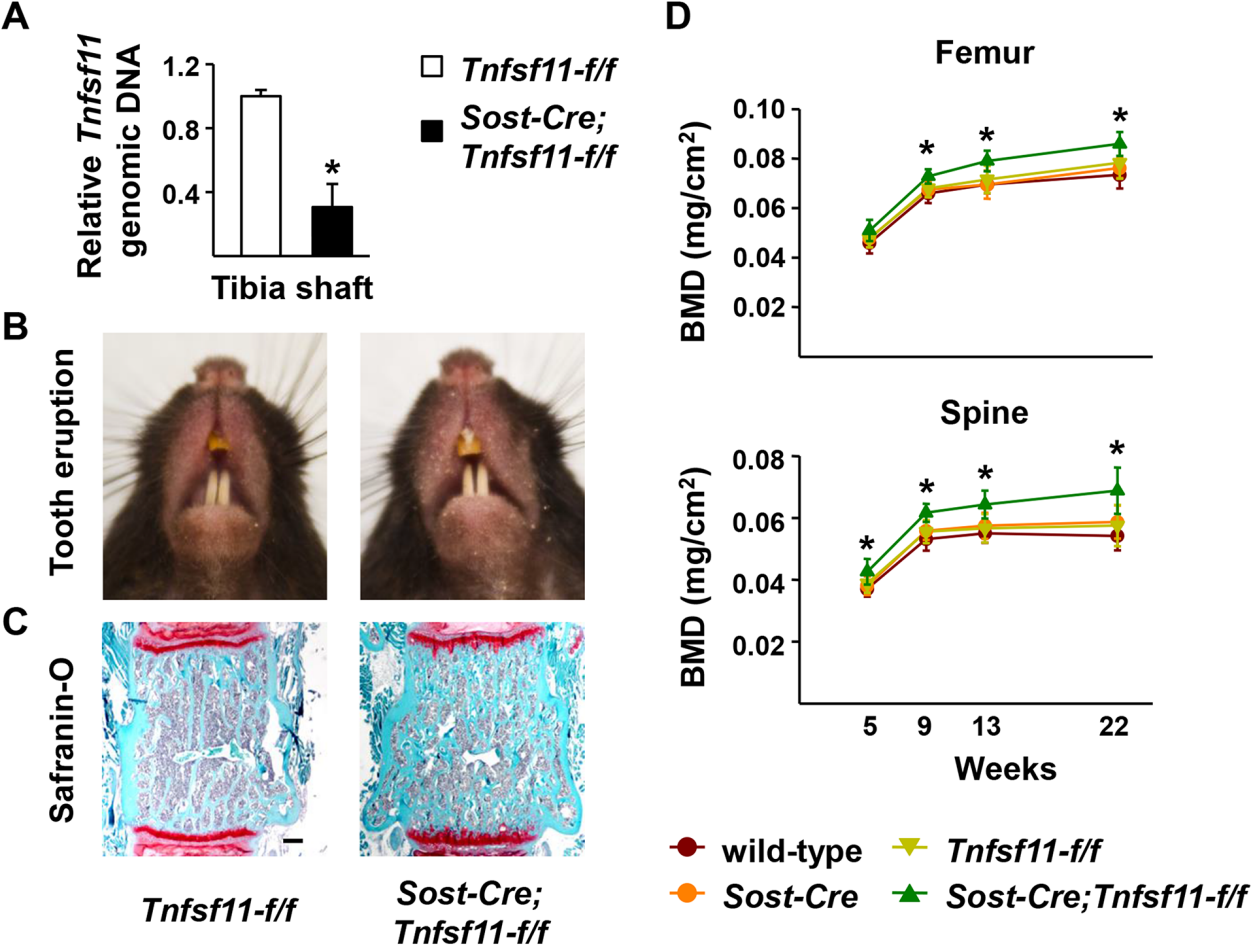
Deletion of *Tnfsf11* using the *Sost-Cre* transgene increases bone mass. (A) Quantitative PCR of loxP-flanked *Tnfsf11* genomic DNA using genomic DNA isolated from collagenase-digested femoral cortical bone of 6-month-old *Sost-Cre;Tnfsf11-f/f* (n = 10) and *Tnfsf11-f/f* (n = 16) littermates. *P < 0.05 using Student’s t-test. Representative images of the teeth (B) and safranin-O-stained histological sections of the lumbar vertebra (Scale bar, 0.1 mm) (C) of 6-month-old *Sost-Cre;Tnfsf11-f/f* and *Tnfsf11-f/f* mice. (D) Serial femoral and spinal BMD of *Sost-Cre;Tnfsf11-f/f* (n = 14), *Tnfsf11-f/f* (n = 16), wild-type (n = 14), and *Sost-Cre* (n = 13) littermates measured by DXA. *P < 0.05 versus wild-type, *Tnfsf11-f/f*, or *Sost-Cre* mice using one-way ANOVA comparing the 4 genotypes at a given age. All results include data from both male and female mice.

**Fig 6:**
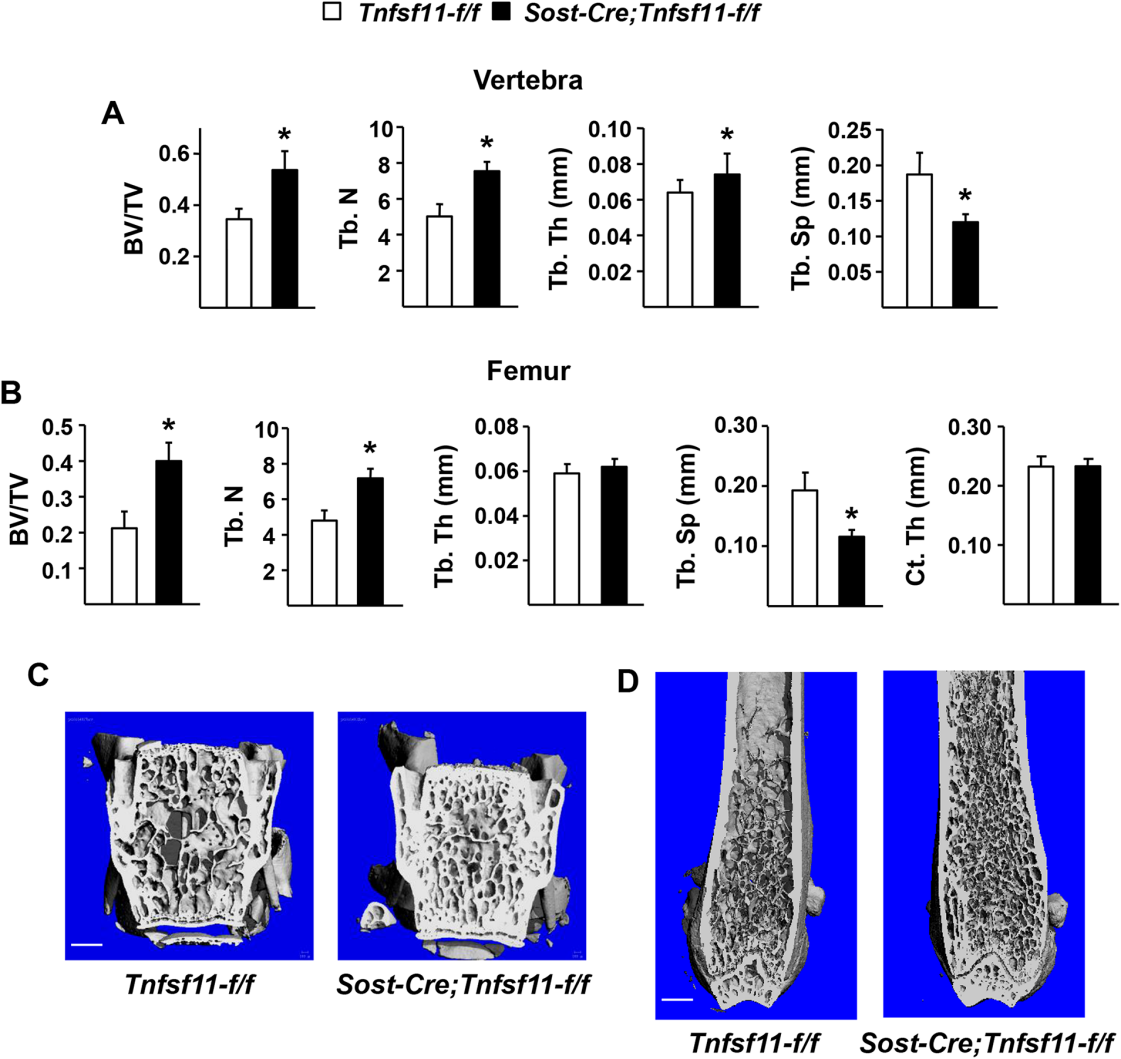
Cancellous bone volume is high in *Sost-Cre;Tnfsf11-f/f* mice. (A) Cancellous bone volume over total volume (BV/TV), trabecular number (Tb.N), trabecular thickness (Tb.Th), and trabecular separation (Tb.Sp) in the L4 vertebra of 6-month-old *Sost-Cre;Tnfsf11-f/f* (n = 10) and *Tnfsf11-f/f* (n = 16) littermates. *P < 0.05 using Student’s t-test. (B) Cancellous BV/TV, trabecular number, trabecular thickness, trabecular separation, and cortical thickness (Ct.Th) in the distal femur of the same mice as in (A). *P < 0.05 using Student’s t-test. Representative μCT images of the L4 vertebra (C) and distal femur (D) of 6-month-old *Sost-Cre;Tnfsf11-f/f* and *Tnfsf11-f/f* littermates. All results include data from both male and female mice. Scale bar, 0.5 mm.

*Tnfsf11-f/f* mice were crossed with *Sost-Cre* mice to delete the *Tnfsf11* gene from osteocytes but not osteoblasts and lining cells. Deletion of the *Tnfsf11* gene was confirmed by quantitative real time PCR using genomic DNA isolated from osteocyte-enriched cortical bone (Fig 5A). *Sost-Cre;Tnfsf11-f/f* mice had normal tooth eruption (Fig 5B) and normal resorption of calcified cartilage during growth as revealed by safranin O staining of cartilage in the lumbar vertebra (Fig 5C). However, spinal bone mineral density (BMD) measured by DXA was increased in *Sost-Cre;Tnfsf11-f/f* mice compared with control littermates beginning at 5 weeks of age (Fig 5D). This difference gradually increased up to at least 22 weeks of age (Fig 5D). Consistent with increased BMD, μCT analysis of L4 vertebra revealed high bone mass in 22-week-old *Sost-Cre;Tnfsf11-f/f* mice and quantification of the cancellous bone compartment revealed high bone volume over total volume, trabecular number, trabecular thickness, and low trabecular separation (Fig 6A and 6C). Similar results were obtained in the cancellous bone of the femur, except that trabecular thickness was not increased (Fig 6B and 6D). Cortical thickness was not affected in *Sost-Cre;Tnfsf11-f/f* mice (Fig 6B).

**Fig 7:**
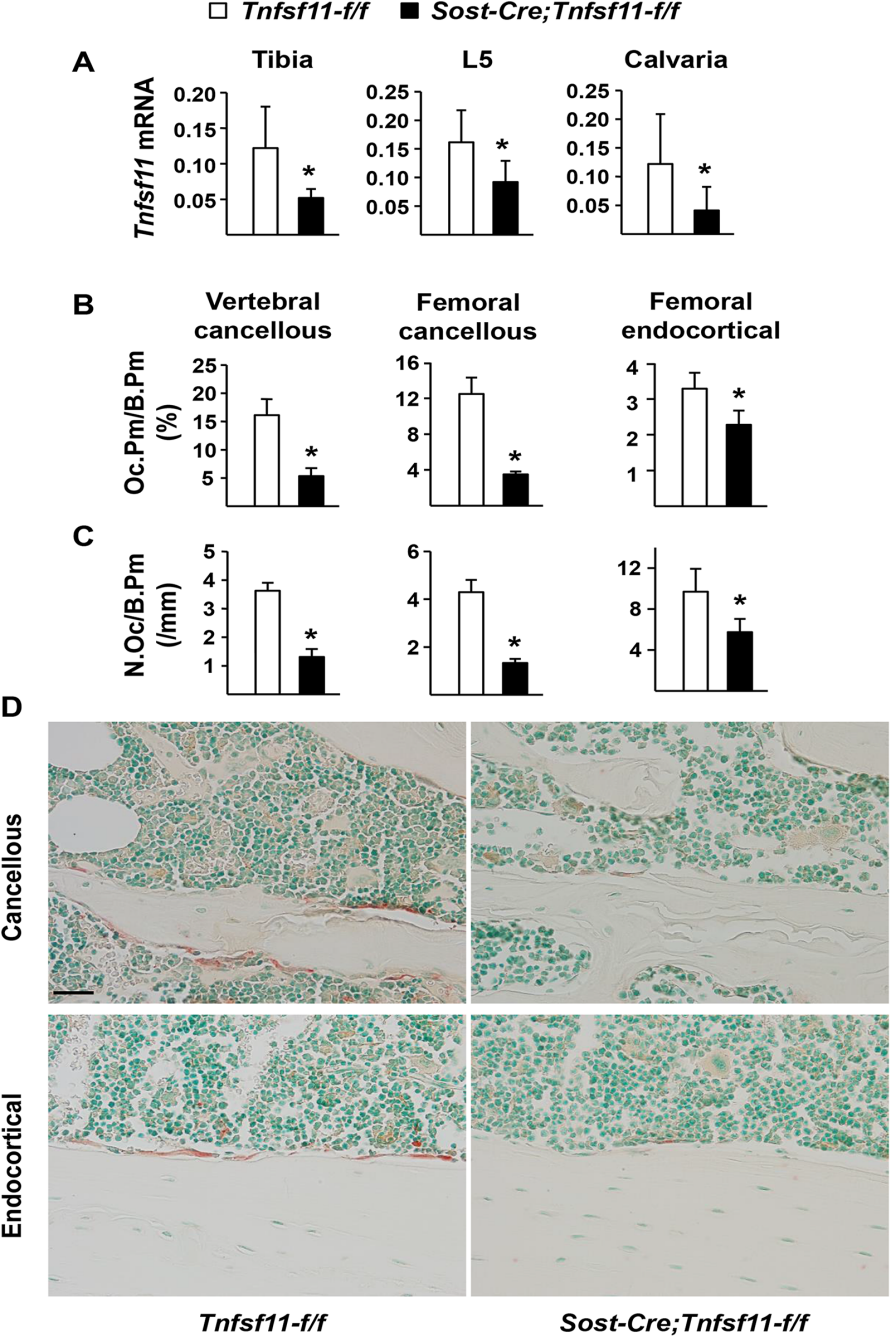
Osteoclastogenesis is inhibited in *Sost-Cre;Tnfsf11-f/f* mice. (A) Quantitative RT-PCR for *Tnfsf11* mRNA in tibia, L5 vertebra, and calvaria of 6-month-old *Sost-Cre;Tnfsf11-f/f* (n = 10) and *Tnfsf11-f/f* (n = 16) littermates. *P < 0.05 using Student’s t-test. (B-C) The perimeter of bone surface covered by osteoclasts (Oc.Pm/B.Pm) (B) and the osteoclast number per mm of bone surface (N.Oc/B.Pm) (C) in cancellous bone of the vertebra (left panel) and distal femur (middle) and endocortical bone (right) of 6-month-old *Sost-Cre;Tnfsf11-f/f* (n = 5) and *Tnfsf11-f/f* (n = 5) littermates. *P < 0.05 using Student’s t-test. (D) Representative images of osteoclasts in cancellous bone (upper panel) and endocortical surface (lower panel) of 6-month-old *Sost-Cre;Tnfsf11-f/f* and *Tnfsf11-f/f* mice. All results include data from both male and female mice.

Consistent with the changes in bone mass and architecture, *Tnfsf11* gene expression was significantly lower in the tibia, L5 vertebra, and calvaria of conditional knockout mice compared to control littermates (Fig 7A and S4 Fig). Moreover, histomorphometric analysis showed that osteoclast number and osteoclast surface were notably lower in cancellous bone of femur and lumbar vertebra of *Sost-Cre;Tnfsf11-f/f* mice (Fig 7B–7D). Osteoclast number and osteoclast surface on the endocortical surface were also reduced in conditional knockout mice, but to a lesser extent than in cancellous bone (Fig 7B and 7C). All of these structural and cellular changes are indistinguishable from those observed in mice in which the *Tnfsf11* gene was deleted using the *10 kb Dmp1-Cre* transgene [7;8]. Taken together, these findings demonstrate that osteocytes, but not osteoblasts or lining cells, provide the majority of the RANKL required for cancellous bone remodeling in both the appendicular and axial skeleton in adult mice.

## Discussion

Control of *Tnfsf11* transcription is complex and involves distant regulatory elements [24;25]. This complexity likely reflects the biological necessity for this gene to be expressed by a variety of different cell types and in response to a number of different signals [6;26]. Moreover, the signaling pathways and gene response elements involved in RANKL production differ significantly between different cell types [24;27–30]. Therefore, to understand the mechanisms that underlie either physiological or pathological bone resorption, it is essential to accurately identify the cellular sources of RANKL involved in osteoclast formation at different sites and under different conditions. For example, since RANKL production by T lymphocytes is not required for the bone loss caused by estrogen deficiency [31], it would be illogical to study estrogen control of the *Tnfsf11* gene in this cell type. Similarly, numerous in vitro studies have focused on production of RANKL by bone marrow stromal cells or cultures rich in osteoblastic cells (reviewed in [6]). Whether results obtained in such cell types accurately reflect mechanisms involved in RANKL production by osteocytes remains unclear.

Immunohistochemical studies of human and murine bone suggest that osteocytes express sclerostin, but osteoblasts and lining cells do not [15]. The activity of the *Sost-Cre* transgene generated for the current study is consistent with this histologic evidence in that osteocytes, but not osteoblasts or lining cells, exhibited reporter gene activation by the transgene. In addition to osteocytes, the *Sost-Cre* transgene also led to recombination in hematopoietic cells. Although this property will limit its general utility, this transgene is nonetheless useful for distinguishing between osteocytes versus osteoblasts and lining cells as sources of RANKL. Previous studies clearly demonstrate that deletion of *Tnfsf11* from cells targeted by the *Dmp1-Cre* transgene potently suppresses cancellous osteoclast formation [7;8]. Similar results were obtained using the *Sost-Cre* transgene. The results of our Cre-reporter analysis show that the only cell type clearly targeted by both transgenes is the osteocyte. Thus, the results of these two studies taken together indicate that osteocytes are the major source of RANKL for cancellous osteoclast formation. Consistent with this contention, deletion of *Tnfsf11* from B and T lymphocytes, which express the highest levels of RANKL within the hematopoietic lineages, does not alter bone mass or osteoclast number under normal physiological conditions [8;31], whereas deletion of *Tnfsf11* using the *Sost-Cre* transgene, which targets osteocytes as well as hematopoietic cells, increases bone mass and reduces osteoclast number.

The main conclusion of our study, and all studies using Cre-loxP approaches, relies on the cell-type specificity of the Cre-deleter strains that were used. We have determined the specificity of *Sost-Cre* mice using two different Cre-reporter lines. One limitation of this approach is that different loxP-flanked alleles can vary in the efficiency of recombination in response to the same Cre-deleter strain [32]. However, the similarities we observed with two independent Cre-reporter models, both of which are highly sensitive to Cre-mediated recombination, strongly suggests that they reflect the same cell populations in which the *Tnfsf11* gene was deleted.

An alternative approach to identify cell types in which *Tnfsf11* was deleted would have been to directly measure RANKL abundance in osteocytes versus osteoblasts and lining cells in both the conditional knockout and control mice. Such studies would require a highly specific antibody. We have screened several anti-RANKL antibodies from different manufacturers and found only one that produces a signal by immunochemistry that is abolished in mice lacking RANKL [7]. However, the affinity of this antibody is low and produces specific signal only in cells that abundantly express RANKL such as hypertrophic chondrocytes [7;33]. Thus we have not been able to detect specific signal in either osteoblasts or osteocytes using this antibody (not shown).

Due to their location at the interface between hematopoietic progenitors in the bone marrow and the bone surface, lining cells have been proposed as a cell type that initiates the process of bone remodeling by recruiting osteoclast progenitors [34]. Moreover, lining cells are thought to contribute to the formation of so-called “canopies” that separate bone remodeling units from the bone marrow [35]. However, the similar reductions in osteoclast number in *Sost-Cre;Tnfsf11-f/f* and *Dmp1-Cre;Tnfsf11-f/f* mice, together with the finding that the *Sost-Cre* transgene does not lead to Cre-mediated recombination in lining cells, argues against a significant contribution of lining cell-derived RANKL in physiological bone remodeling.

We have shown previously that the increase in osteoclast formation and bone loss caused by either hind limb unloading or dietary calcium deficiency are inhibited in *Dmp1-Cre;Tnfsf11-f/f* mice [7;36]. Moreover, unloading and dietary calcium deficiency stimulated the levels of *Tnfsf11* mRNA in osteocyte-enriched bone from mice with the control genotype (*Tnfsf11-f/f*). Based on this evidence, we had concluded that osteocyte-derived RANKL is also important for these types of pathological bone resorption. However, since the *Tnfsf11* gene was deleted from osteoblasts and lining cells, as well as osteocytes, in these earlier studies, the *Sost-Cre* transgenic mice generated in the present report should be useful to address a potential contribution of osteoblast-derived RANKL in these and other pathological conditions that lead to bone loss.

Deletion of *Tnfsf11* with the *Sost-Cre* transgene in the present work reduced endocortical osteoclast number by approximately 30%, which is a much smaller effect than the approximately 70% reduction in osteoclast number in cancellous bone. A similar finding was observed previously after deletion of *Tnfsf11* with the *10 kb Dmp1-Cre* transgene [36]. These results suggest that a significant amount of the RANKL involved in osteoclast formation at the endocortical surface is not produced by osteocytes, osteoblasts, lining cells, or hematopoietic cells. Other potential sources of RANKL for this process include osteoblast-lineage cells at a stage prior to expression of the *10 kb Dmp1-Cre* transgene or bone marrow stromal cells not of the osteoblast lineage. Alternatively, osteoclast generation in the cancellous versus endocortical surface may be differentially influenced by other osteoclastogenic agents acting synergistically with RANKL, for example Wnt5A [37]. Nonetheless, osteocyte-derived RANKL contributes significantly to pathological bone resorption at this site since the increase in endocortical osteoclast number caused by dietary calcium deficiency was blunted in *10 kb Dmp1-Cre;Tnfsf11-f/f* mice [36], although a contribution by osteoblasts and lining cells cannot be excluded. Similarly, RANKL produced by B lymphocytes plays no role in normal bone remodeling but is essential for the increase in osteoclast number in cancellous bone caused by estrogen deficiency [31] These findings demonstrate that the relative importance of different cellular sources of RANKL can change depending on physiological conditions.

In conclusion, we have provided new evidence that RANKL produced by osteocytes is required for osteoclast formation in cancellous bone. It will be important in future studies to determine how RANKL produced by osteocytes is able to bind to osteoclast progenitors in the bone marrow and whether all osteocytes or a subset of osteocytes are involved. A better understanding of the molecular mechanisms that control *Tnfsf11* gene expression in osteocytes, as well as other cell types, may be useful for the development of therapeutic approaches to specifically suppress pathological bone resorption.

## Supporting Information

S1 Fig Comparison of *10 kb Dmp1-Cre* and *8 kb Dmp1-Cre* mice using *R26R* Cre-reporter mice. Brightfield (A, C, and E) and epifluorescence (B, D, and F) microscopy images of X-gal stained frozen histological sections of cancellous bone (A-D) and cortical bone (E-F) in the femur of 2-month-old *Sost-Cre;R26R*, *10 kb Dmp1-Cre;R26R*, and *R26R* mice. X-gal deposits are blue in the brightfield images. The images in B, D, and F are epifluorescence images of the same sections shown in A, C, and E, respectively, and show calcein labeling (green). Scale bar, 50 μm.
(PPTX)

S2 Fig Fluorescent cells on the bone surface of *Sost-Cre;tdTomato* mice are osteoclasts. Epifluorescence and brightfield microscopy images of frozen histological sections of femoral cancellous bone from 2-month-old *tdTomato*, *Sost-Cre;tdTomato*, and *10 kb Dmp1-Cre;tdTomato* mice. Images on the left are epifluorescence showing tdTomato-positive cells in bone (osteocytes) as well as on the bone surface. Images on the right are brightfield images of the same sections after staining for TRAP activity (red), showing that the tdTomato-positive cells in *Sost-Cre;tdTomato* mice are osteoclasts but that the tdTomato-positive cells in *Dmp1-Cre;tdTomato* mice are not.
(PPTX)

S3 Fig Hematopoietic lineage analysis of bone marrow cells from *Sost-Cre;tdTomato* mice by flow cytometry. (A) Flow cytometry data plot indicating the percentage of total bone marrow cells from the femur of *Sost-Cre;tdTomato* mice that exhibit tdTomato fluorescence. (B) Flow cytometry data plots of bone marrow cells obtained from the femur of *Sost-Cre;tdTomato* mice and stained with the indicated antibodies. Plots on the top are for total bone marrow cells whereas plots on the bottom represent only the tdTomato-positive fraction.
(PPTX)

S4 Fig *Tnfsf11* expression in skeletal tissues. Quantitative RT-PCR for *Tnfsf11* mRNA in tibia, L5 vertebra, and calvaria of 6-month-old *Sost-Cre;Tnfsf11-f/f* (n = 10) and *Tnfsf11-f/f* (n = 16) littermates. Normalized to *β-actin*. *P < 0.05 using Student’s t-test.
(PPTX)
