# Supplementary figures and images for: Osteocytes, not Osteoblasts or Lining Cells, are the Main Source of the RANKL Required for Osteoclast Formation in Remodeling Bone
Source: PLoS One. 2015 Sep 22;10(9):e0138189. doi: 10.1371/journal.pone.0138189 (PMC4578942; doi:10.1371/journal.pone.0138189)

## Slide 1
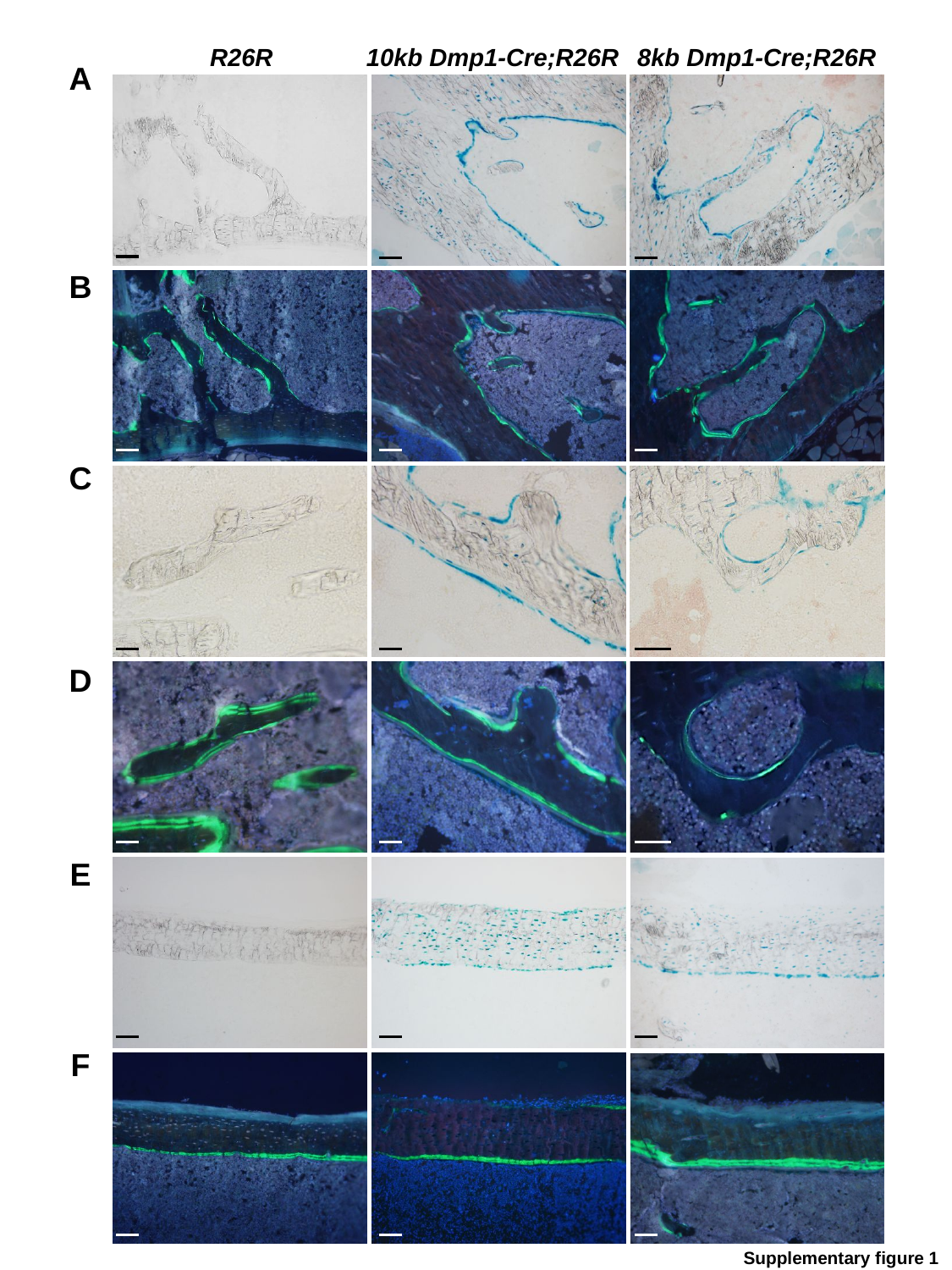

R26R
10kb Dmp1-Cre;R26R
8kb Dmp1-Cre;R26R
A
B
C
D
E
F
Supplementary figure 1

Supplement: S1 Fig — Brightfield (A, C, and E) and epifluorescence (B, D, and F) microscopy images of X-gal stained frozen histological sections of cancellous bone (A-D) and cortical bone (E-F) in the femur of 2-month-old Sost-Cre;R26R, 10 kb Dmp1-Cre;R26R, and R26R mice. X-gal deposits are blue in the brightfield images. The images in B, D, and F are epifluorescence images of the same sections shown in A, C, and E, respectively, and show calcein labeling (green). Scale bar, 50 μm. (PPTX) [file pone.0138189.s001.pptx]

## Slide 1
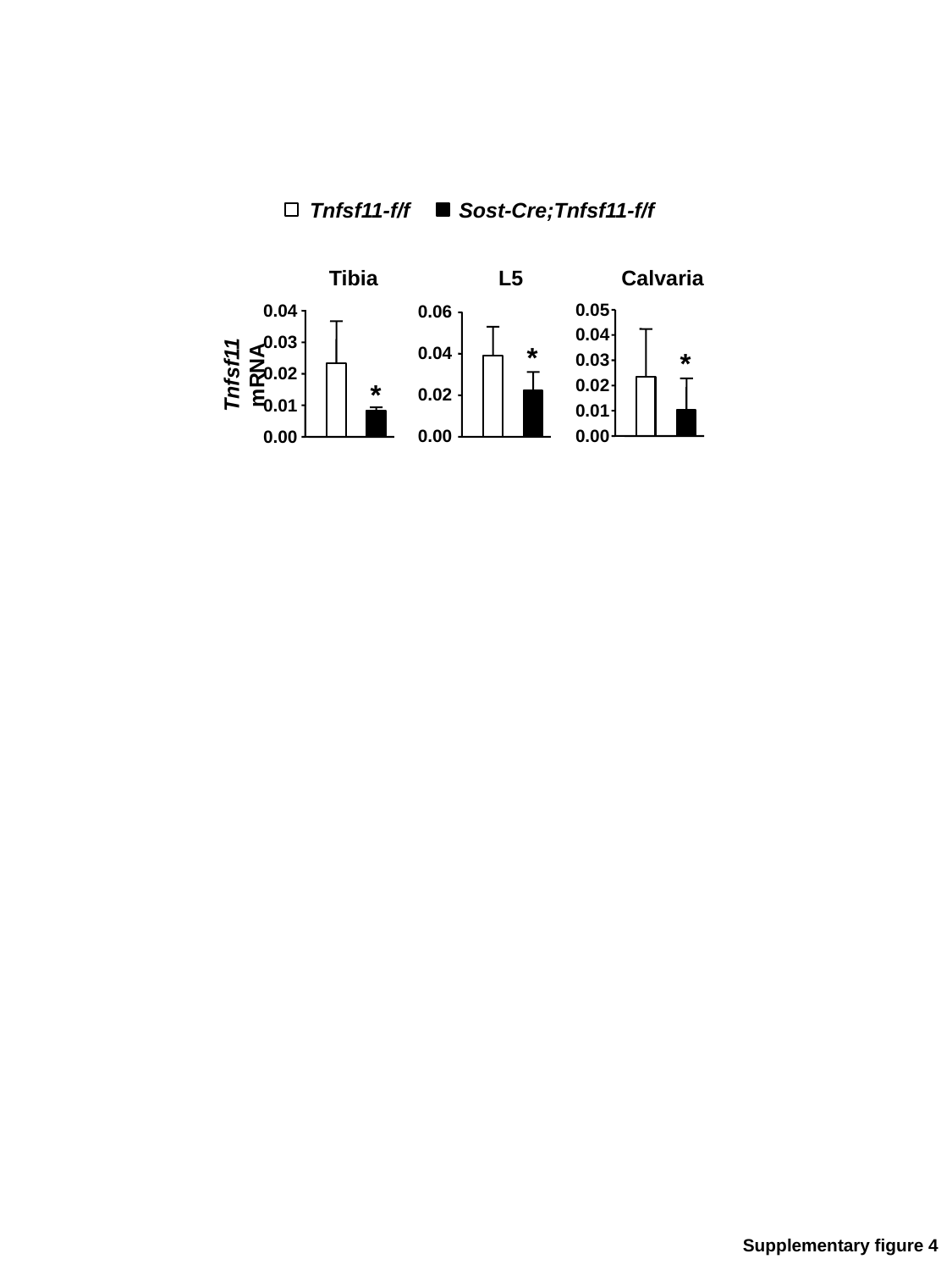

Tnfsf11-f/f
Sost-Cre;Tnfsf11-f/f
Tibia
L5
Calvaria
Tnfsf11 mRNA
0.05
0.04
0.06
0.04
0.03
*
*
0.04
0.03
0.02
*
0.02
0.02
0.01
0.01
0.00
0.00
0.00
Supplementary figure 4

Supplement: S4 Fig — Quantitative RT-PCR for Tnfsf11 mRNA in tibia, L5 vertebra, and calvaria of 6-month-old Sost-Cre;Tnfsf11-f/f (n = 10) and Tnfsf11-f/f (n = 16) littermates. Normalized to β-actin. *P < 0.05 using Student’s t-test. (PPTX) [file pone.0138189.s004.pptx]
