# Supplementary material for: Osteocytes, not Osteoblasts or Lining Cells, are the Main Source of the RANKL Required for Osteoclast Formation in Remodeling Bone
Source: PLoS One. 2015 Sep 22;10(9):e0138189. doi: 10.1371/journal.pone.0138189 (PMC4578942; doi:10.1371/journal.pone.0138189)
Supplement: S2 Fig — Epifluorescence and brightfield microscopy images of frozen histological sections of femoral cancellous bone from 2-month-old tdTomato, Sost-Cre;tdTomato, and 10 kb Dmp1-Cre;tdTomato mice. Images on the left are epifluorescence showing tdTomato-positive cells in bone (osteocytes) as well as on the bone surface. Images on the right are brightfield images of the same sections after staining for TRAP activity (red), showing that the tdTomato-positive cells in Sost-Cre;tdTomato mice are osteoclasts but that the tdTomato-positive cells in Dmp1-Cre;tdTomato mice are not. (PPTX) [file pone.0138189.s002.pptx]

## Slide 1
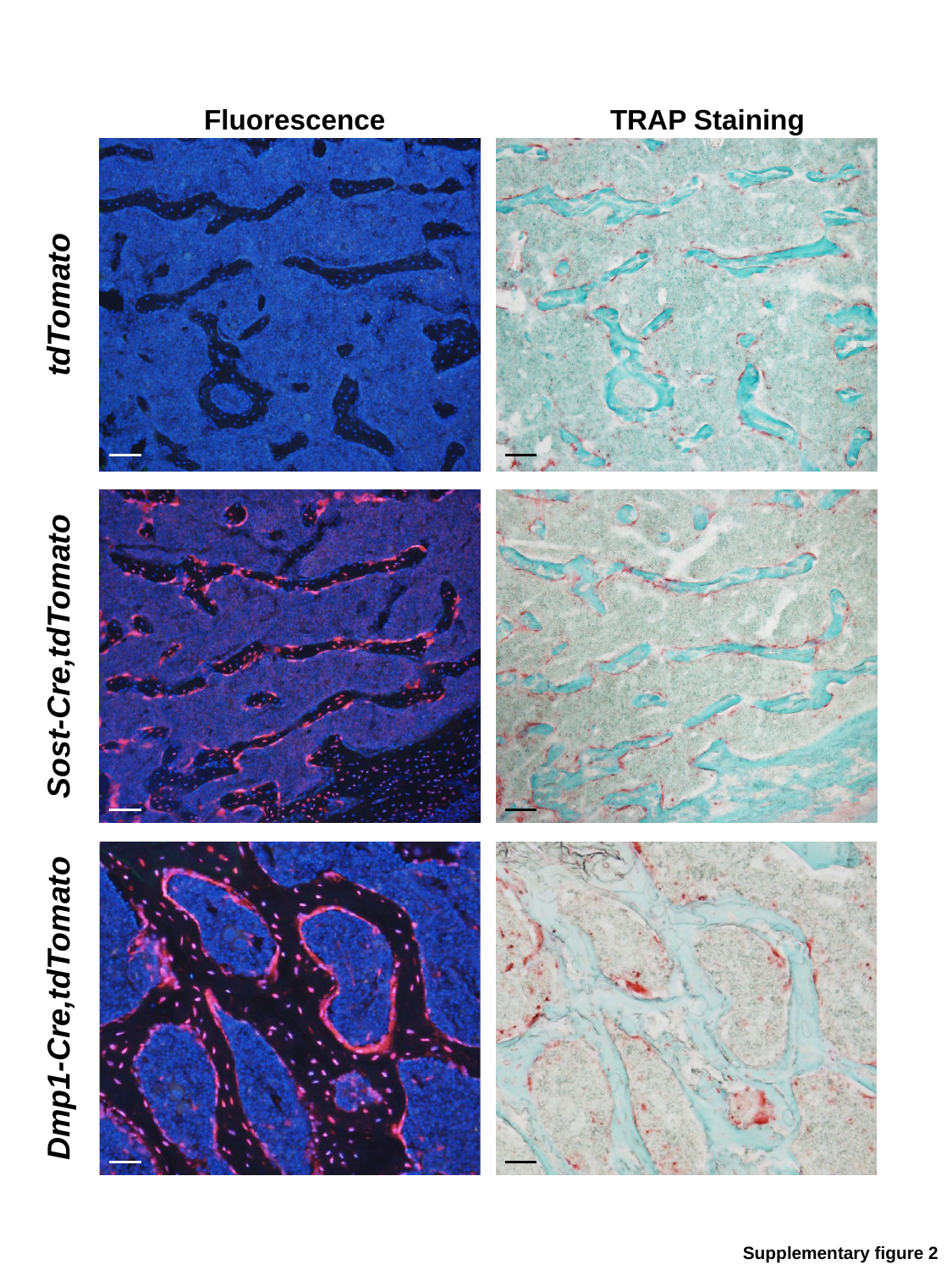

Fluorescence
TRAP Staining
tdTomato
Sost-Cre,tdTomato
Dmp1-Cre,tdTomato
Supplementary figure 2
