# Supplementary material for: Osteocytes, not Osteoblasts or Lining Cells, are the Main Source of the RANKL Required for Osteoclast Formation in Remodeling Bone
Source: PLoS One. 2015 Sep 22;10(9):e0138189. doi: 10.1371/journal.pone.0138189 (PMC4578942; doi:10.1371/journal.pone.0138189)
Supplement: S3 Fig — (A) Flow cytometry data plot indicating the percentage of total bone marrow cells from the femur of Sost-Cre;tdTomato mice that exhibit tdTomato fluorescence. (B) Flow cytometry data plots of bone marrow cells obtained from the femur of Sost-Cre;tdTomato mice and stained with the indicated antibodies. Plots on the top are for total bone marrow cells whereas plots on the bottom represent only the tdTomato-positive fraction. (PPTX) [file pone.0138189.s003.pptx]

## Slide 1
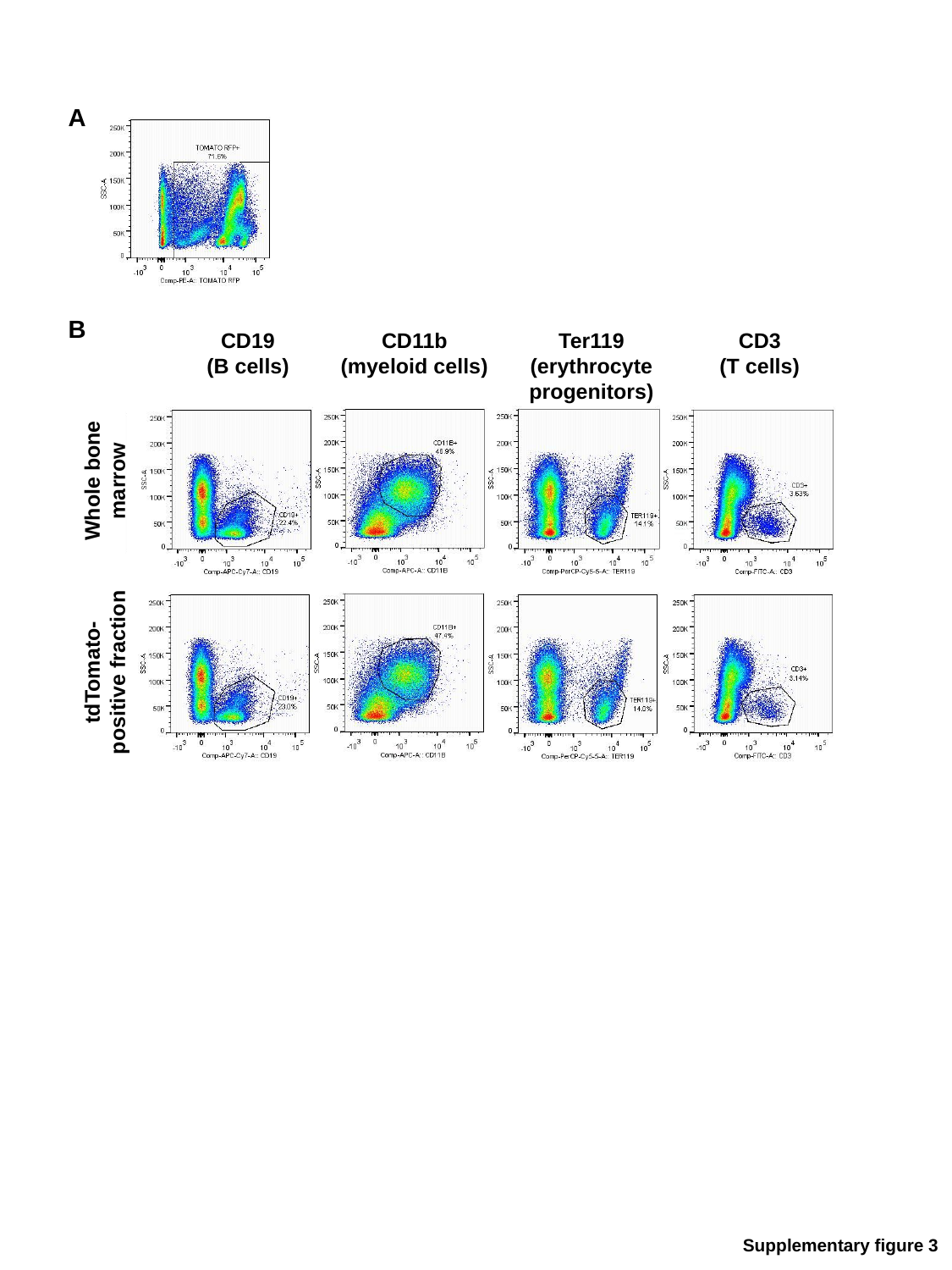

A
B
CD19
(B cells)
CD11b
(myeloid cells)
Ter119
(erythrocyte
progenitors)
CD3
(T cells)
Whole bone
marrow
tdTomato-positive fraction
Supplementary figure 3
